# Effect of chemical surface treatments on the repair of composite restorations

**DOI:** 10.4317/jced.63057

**Published:** 2025-10-01

**Authors:** Fabio Rizzante, Hannah Hales, Italo Silva, Monica Cayouette, Sergio Ishikiriama, Adilson Furuse

**Affiliations:** 1 DDS, MSc, PhD, MBA. Department of Reconstructive and Rehabilitation Sciences, James B. Edwards College of Dental Medicine, Medical University of South Carolina, Charleston, SC, USA; 2 DDS, MSc, PhD, MBA. Private Practice, Charleston, SC, USA; 3 BSc, PhD. Case Western Reserve University, Cleveland, OH, USA; 4 DMD, MSc, Department of Reconstructive and Rehabilitation Sciences, James B. Edwards College of Dental Medicine, Medical University of South Carolina, Charleston, SC, USA; 5 DDS, MSc, PhD, Department of Operative Dentistry, Dental Materials and Endodontics, Bauru School of Dentistry, University of São Paulo, Bauru, SP, Brazil

## Abstract

**Background:**

Repair of resin composite restorations consists in a more conservative solution compared to complete replacement. The objective of the present study was to evaluate different surface treatment protocols and their effects on the adhesive interface between the base and the repair resin composite, considering both new and aged restorations.

**Material and Methods:**

This study evaluated six resin composites (Admira Fusion Xtra/ADM, Filtek Supreme Flowable/FSF, Filtek One/FO, Vitra/VIT, Filtek Supreme/FS, Filtek Bulk Fill Flowable/FBF), five surface treatments (Hydrofluoric acid 120s+silane/HFs, Phosphoric acid 30s/P30, Phosphoric acid 30s+silane/P30s, Sof-lex+phosphoric acid 30s/SP30, Sof-lex+phosphoric acid 30s+silane/SP30s), and two repair timepoints (immediate and after 1 year simulated aging aged). Forty disks for each resin were divided into 10 groups according to surface treatment and repair timepoints (*n*=4 disks per subgroup). Surface treatments were performed, followed by application of a universal bonding agent (Scotchbond Universal). Filtek Supreme/FS was used as the repairing resin, and three cylinders of material were cemented on each resin disk. Notched shear bond strength test was performed using a universal testing machine, contact angle tests were performed using a goniometer, and fracture mode analysis was performed using a stereomicroscope.

**Results:**

All factors and their interactions were significant for both shear bond and contact angle tests (*p*<.001 for all criteria). Overall results for shear bond strength showed SP30=SP30s>P30=P30s>HFs; immediate<aged; and FS=FO>VIT>FBF=FSF>ADM. Similarly, overall results for contact angle showed P30>HFs>SP30; aged>immediate; and FSF>VIT=FBF>FS=FO>ADM. Immediate resin samples treated with mechanical roughening exhibited lower number of adhesive failures compared to other treatments. In aged samples, groups treated with mechanical roughening and/or silane agent showed a predominance of cohesive and/or mixed failure modes. Shear bond strength is influenced by the type and age of the base resin composite, as well as the surface treatment applied.

**Conclusions:**

Despite a tendency for higher results when mechanical roughening is associated with Scotchbond Universal, there is not a clear difference to justify its use in most of the resin composites. Furthermore, most of the surface treatments performed similarly, regardless of the base resin composite.

** Key words:**Resin composite, resin repair, surface treatments, bulk-fill composite, adhesive interface.

## Introduction

New materials are constantly being developed for the dental field. Among them, resin composites can be considered one of the most versatile restorative materials available to clinicians, indicated for direct, semi-direct, and indirect restorations in both anterior and posterior teeth [1-6]. The objectives of such new developments include providing fast, reliable, conservative, long-lasting, and technically simpler treatments. Within this context, bulk-fill resin composites were introduced to the market with the purpose of allowing faster restorations due to the possibility of using larger increments based on their lower polymerization shrinkage and increased depth of cure [1,2]. Such composites are capable of reducing the number of clinical steps and simplifying the restorative technique when compared with the incremental technique using regular composites, while providing similar clinical results [1,2].

Despite regular and bulk-fill resin composites being extensively used and supported by the literature, the substitution of resin composite restorations continues to occur mainly due to marginal degradation, secondary decay and fractures, necessitating some form of intervention. Other than the complete removal and replacement of defective restorations, the repair of composite restorations is a more conservative, cost-effective, and time-efficient approach, compatible with the concepts of minimally invasive dentistry [4-14].

A long-lasting repair will depend on the quality of the remaining resin composite, including decontaminating its surface with surface roughening or acid conditioning, the quality of the adhesive interface between the base and the new/repairing composite, and the new composite itself [3-6,8,10,11,13,15-22]. Despite the well-known fact that resin composites degrade when exposed to the oral environment, it is not clear if, or how, this degradation may jeopardize the repairing capabilities of different materials [4,5,11].

Considering the widely variable composition of resin composites, it is unclear which surface treatments could best promote bonding between the fractured resin and the composite used for repair. Nevertheless, it is expected that increasing the surface roughness through the use of strong acids such as hydrofluoric acid, or surface abrasion, would provide better mechanical interlocking at the repair interface [4-6,8,10,13-22].

Despite advancements in bonding techniques and the development of simplified and universal bonding agents containing 10-methacryloyloxydecyl dihydrogen phosphate (MDP) and silane (e.g., Single Bond Universal), it remains unclear if combinations of surface treatments and/or additional application of silane agents could provide a more reliable adhesive interface, especially when more recent materials are considered, such as bulk-fill resins [3,5,7,11,22]. Thus, the objective of the present study was to evaluate different surface treatment protocols (five levels) and their effects on the adhesive interface between the base (six different composites) and the repair resin, considering both new (seven days’ storage) and aged restorations (one-year simulated aging).

The null hypothesis tested were:

1- There would be no differences in the adhesive interface between base and repair resin 

2- There would be no differences in the adhesive interface considering aging time points for the base resin

## Material and Methods

The present study evaluated three factors: 1) resin composites in six levels (six different resin composites); 2) surface treatments in five levels; and 3) repair time points in two levels (immediate and aged resin). The response variables were the micro shear bond strength assessed using a universal testing machine, the failure mode examined using a stereomicroscope, and the contact angle of the treated surfaces using a contact angle meter.

The different resin composites and treatment protocols are listed in  Table 1 and  Table 2, respectively. Two hundred forty resin composite disks (8mm diameter x 1.5mm thickness) were prepared using a polyvinylsiloxane (PVS) matrix placed on top of a flat glass plate. After filling the matrix with resin composite, a mylar strip and a second glass plate were placed over the samples, followed by 40 seconds of light curing using a 1000mW/cm2 broad-spectrum LED light curing unit (VALO Grand, Ultradent, South Jordan, UT, US) in contact with the mylar strip.

The cured samples were stored in distilled water at 37ºC for 24 hours, and embedded in self-curing acrylic resin (Ortho-Jet, Lang Dental Manufacturing Co) cylinders (25mm diameter x 20mm height) using a stainless-steel matrix. After 15 minutes from acrylic resin manipulation, the cylinders were removed from the matrix and the samples’ surfaces were polished with 320-, 600- and 1200-grit sandpaper (Leco Corp, St Joseph, MI) for 30 seconds each on a polishing machine (Leco SS200, Leco Corp), under water cooling, to remove any interference of the acrylic resin and standardize the sample surface.

Forty resin disks were prepared for each of the six resin composites under study (*n*=40 per group). Resin groups were then subdivided following the five different surface treatments (*n*=8 per subgroup) ( Table 2). Half of the eight samples were stored in distilled water at 37º for seven days, after which the respective surface treatments were applied, followed by another 24 hours of water storage. This represented the “immediate” group. The other half of the samples underwent 10,000 thermal cycles (5°C and 55°C, with a dwell time of 25 seconds at each temperature and a transfer time of five seconds), simulating one-year of intraoral exposure [3], before receiving the surface treatments, which represented the “aged” group.

The surface treatments were performed according to  Table 2. For the HFs subgroup, a 9% hydrofluoric acid (Porcelain Etch, Ultradent) was applied to the sample surfaces for two minutes, followed by rinsing with air/water spray. For subgroups using phosphoric acid (P30, P30s, SP30, and SP30s), 37% phosphoric acid was applied to the sample surfaces for 30 seconds, followed by rinsing with air/water spray

In subgroups using Sof-Lex disks (SP30 and SP30s), surface roughening was conducted by a calibrated operator using the coarsest Sof-Lex disc (3M ESPE, St Paul, MN) at 10,000 RPM. Sof-lex disks were selected as they are commonly available in different markets around the world. The operator applied 10 quick strokes across the resin surface, followed by another 10 strokes perpendicular to the initial ones. Subsequently, all samples were rinsed with air/water spray for 30 seconds and dried with air spray for an additional 30 seconds before the application of 37% phosphoric acid.

For subgroups involving silane application (HFs, P30s and SP30s), a silane agent (RelyX ceramic primer, 3M ESPE) was actively applied over the samples surface using a microbrush during 20s and left undisturbed for one minute to evaporate solvents.

After the respective surface treatments, a universal bonding agent (Scotchbond Universal, 3M ESPE) was applied to the surfaces according to the manufacturer’s recommendations, consisting of application with a rubbing motion, solvent-evaporation with air for five seconds, followed by 20s light curing using the same broad-spectrum LED light curing unit and irradiance previously described. The repairing resin (Filtek Supreme, 3M ESPE) was then applied over the treated surfaces with the aid of a cylindrical Teflon mold (2.38mm diameter x 2mm height) and light-cured for 20 seconds. Three cylinders of repairing resin were placed over each resin disc, resulting in a total of 12 repairing resin samples for each subgroup at each time point.

After the repairing resins were light cured, all samples were stored in distilled water at 37ºC for 24 hours, followed by the notched-edge shear bond test using a Universal testing machine (Ultratester, Ultradent, South Jordan, UT, USA), with a 5000N loading cell. The test was conducted at a crosshead speed of 0.5mm/min in a downward movement until specimens fractured. The fracture load (in MPa) was recorded, and the fractured samples were observed under a stereomicroscope to analyze the fracture mode (adhesive, cohesive, or mixed).

In order to evaluate the effects of surface treatments, excluding the effects of silane and/or adhesive application, an additional nine discs of each resin composite were obtained and treated to represent different subgroups (*n*=3 per subgroup): hydrofluoric acid 120s (group HFs), phosphoric acid 30s (representing groups P30 and P30s), and Sof-Lex + phosphoric acid 30s (representing groups SP30 and SP30s). These samples were then tested for static contact angle using an Optical Contact Angle Meter (CAM200, KSV Instruments), with measuring range between 4-180º with an accuracy of ±1º. Five measurements using a 0.1μm droplet of deionized water over the treated surfaces were performed at room temperature.

Data were subjected to normality test using Kolmogorov-Smirnov test, followed by analysis using 3-way ANOVA and Tukey test for post-hoc analysis (α = 0.05). Additionally, a Pearson correlation test was conducted to explore the interaction between shear bond strength and contact angle.

## Results

The results for shear bond strength can be observed in  Table 3 and  Table 4, while the results for contact angle can be found. All factors (surface treatment, resin type, and time of evaluation) as well as the interactions between these factors were found to be significant for both the shear bond strength and contact angle tests (*p*<.001 for all criteria).

**Figure 1:**
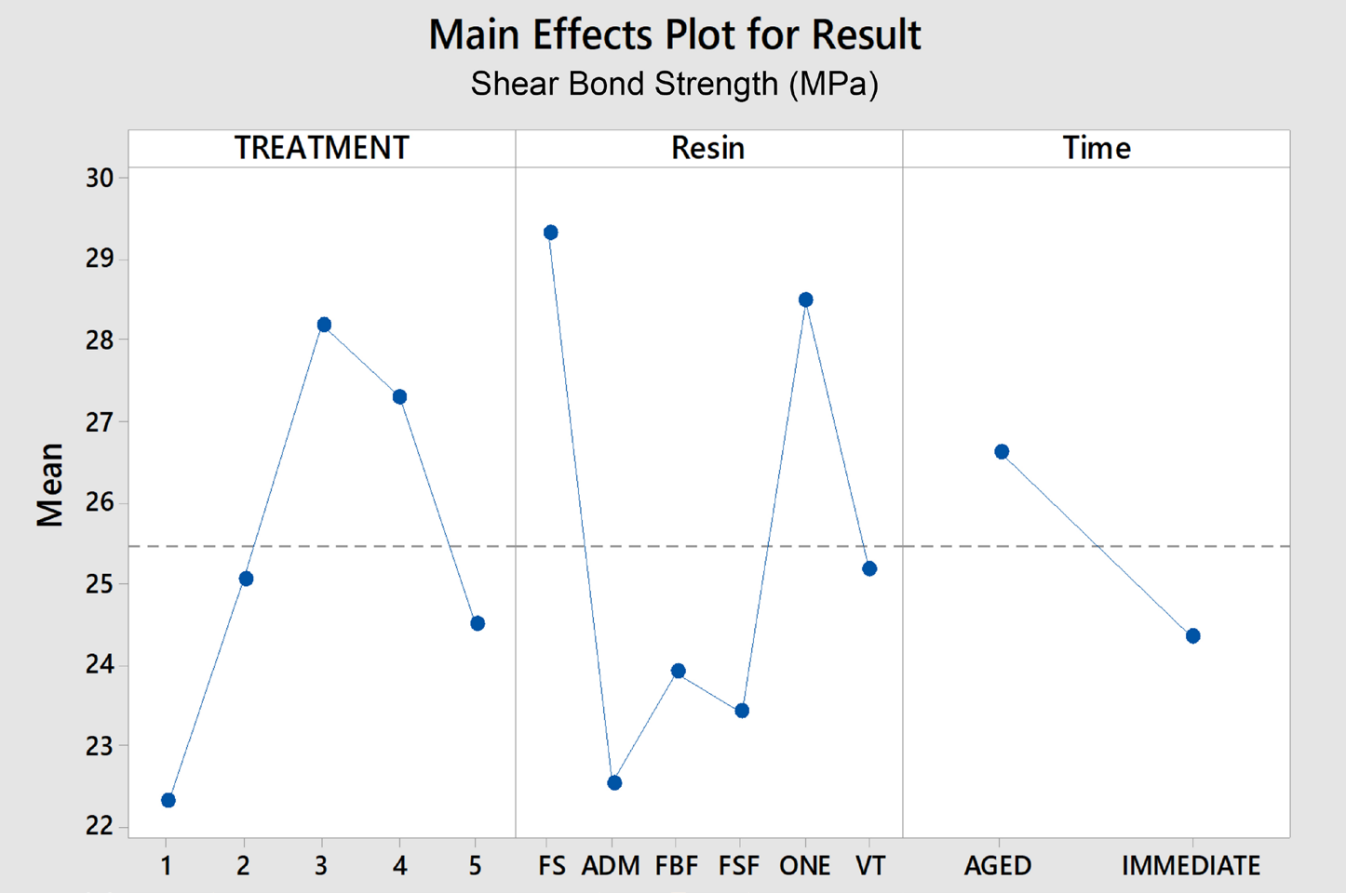
Overall results of Shear Bond Strength, in MPa, considering surface treatment, resin composite and time.

**Figure 2:**
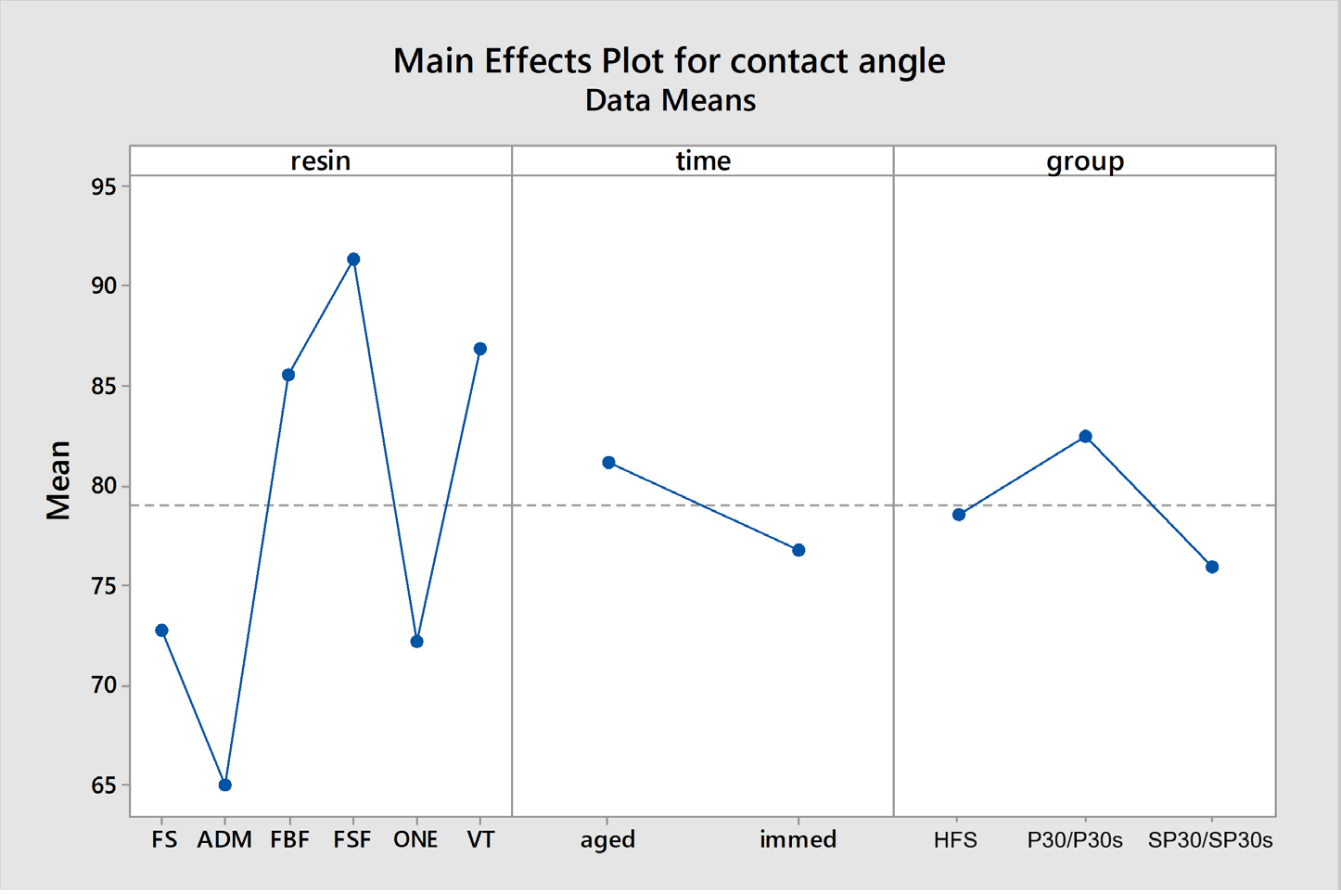
Overall results of Contact Angle considering surface treatment, resin composite and time.

Overall results for shear bond strength (Fig. 1) showed significant differences for surface treatment (*p*<0.001): SP30=SP30s>P30=P30s>HFs; time (*p*<0.001): immediate<aged; and resin type (*p*<0.001): FS =FO>VIT>FBF=FSF>ADM. Similarly, overall results for contact angle (Fig. 2) showed significant differences for surface treatments (*p*<0.001): P30>HF>SP30; time (*p*<0.001): aged>immediate; and for resin type (*p*<0.001): FSF>VIT=FBF>FS=FO>ADM.

For all resins, there was a tendency towards lower shear bond strength values observed in immediate samples treated with hydrofluoric acid (group 1), although this trend was not consistently observed in aged samples (except for ADM, which exhibited even lower values in aged samples). Additionally, there was a tendency towards higher shear bond strength values with treatments involving mechanical surface roughening, either alone or in combination with a silane agent (groups SP30 and SP30s). However, for most resins, these treatments did not significantly differ from other tested protocols.

The exceptions considering immediate resins were: FS (SP30 compared with group HFs); ADM (SP30 compared with P30 and P30s); FBF (P30s was different from HFs, SP30 and SP30s); Filtek Supreme flow (HFs and P30s when compared with P30 and SP30); FO (HFs versus HP30 and SP30; and SP30 versus SP30s and P30s). For aged resin composites, the exceptions were: ADM showed different results when compared with the other groups for HFs; all groups were similar for treatments P30, SP30 and P30s. For SP30s, FS showed differences when compared with all groups but VIT.

Considering the difference between aged and non-aged samples, the bond strength values for composites subjected to artificial aging (10 thousand thermal cycles) tended to be higher when compared with “immediate” samples, although most of the results were similar. Significant differences were observed in FSF and FO for treatment HFs, ADM for SP30, FS for treatment SP30s, and FBF for treatment P30s.

Overall, FS and FO showed the highest shear bond strength, followed by VIT. On the other hand, ADM, FBF, and FSF showed lower overall results. When comparing the same surface treatments, most of the evaluated aged composites exhibited similar behavior. However, exceptions were observed for ADM treated with hydrofluoric acid, which differed from the other resins, and FS treated with SP30s, which exhibited differences compared to the other resins (except VIT).

The effects of surface treatments and time points on contact angle were dependent on the tested resin composites. Treatment with hydrofluoric acid tended to reduce the contact angle for immediate samples compared to aged samples. Additionally, there was a tendency for higher contact angles in aged samples compared to immediate samples, except for ADM and composites treated with mechanical roughening (SP30 and SP30s), except VIT. Furthermore, for all immediate samples, groups SP30 and SP30s usually resulted in an increase in contact angle, except for FS.

**Figure 3:**
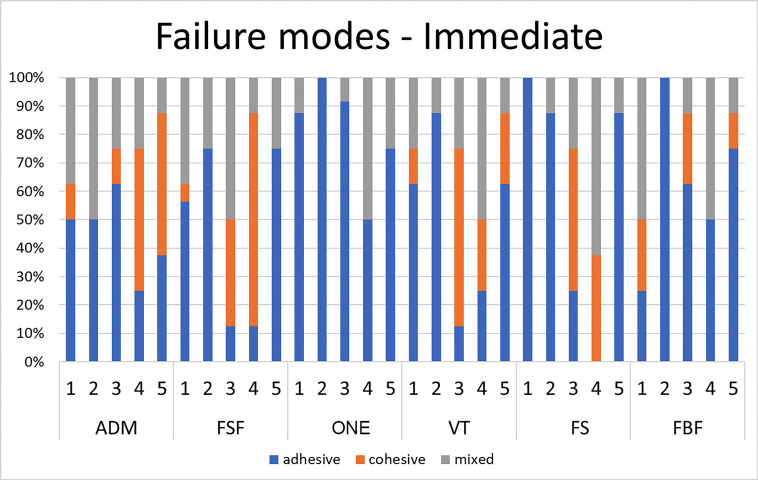
Failure modes for new resin composites.

**Figure 4:**
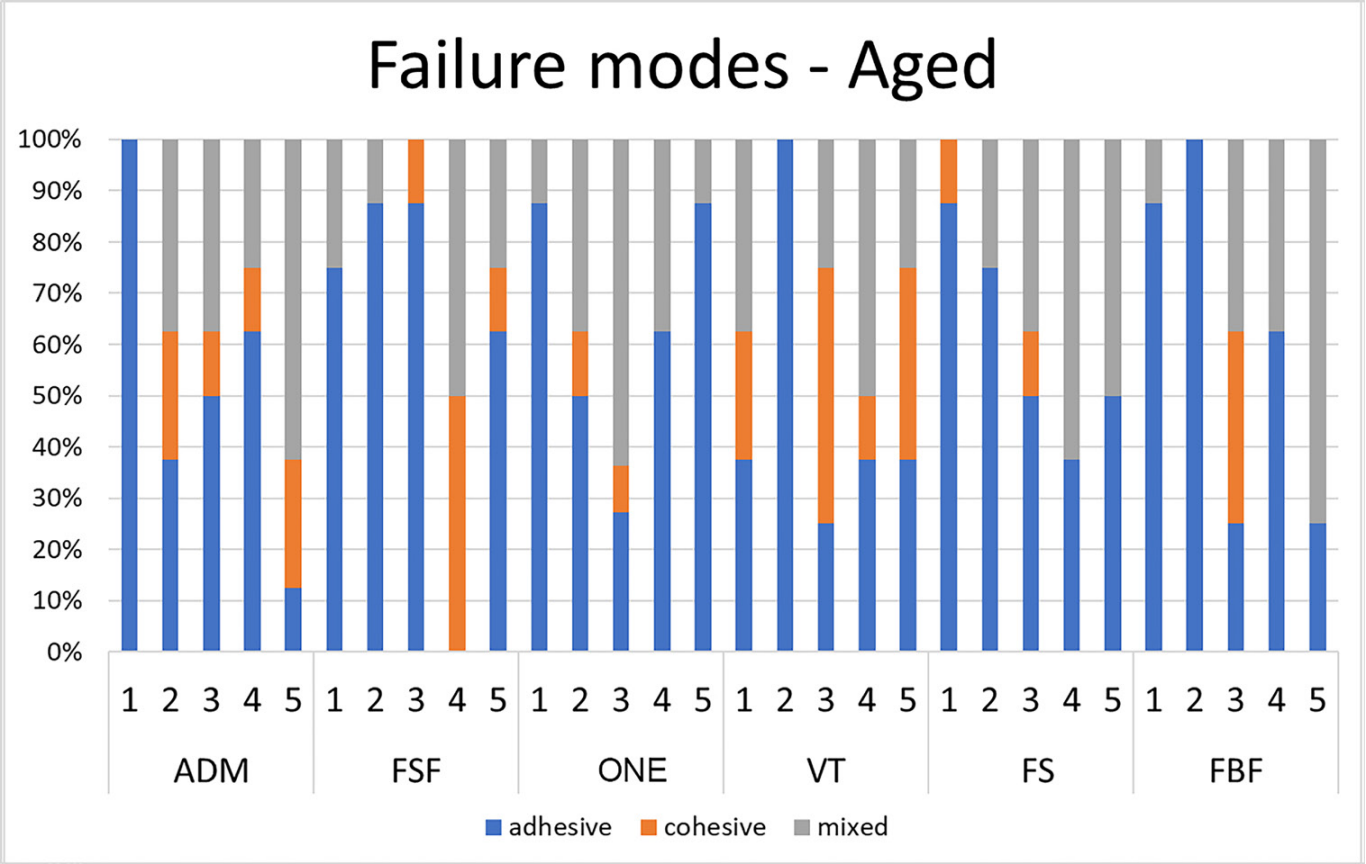
Failure modes for aged resin composites.

The results of fracture mode analysis are depicted in Figures 3 and 4. Overall, immediate resin samples treated with mechanical roughening (SP30 and SP30s) exhibited a lower number of adhesive failures compared to other treatments. In aged samples, groups treated with mechanical roughening and/or silane agent (SP30, SP30s, and P30s) showed a predominance of cohesive and/or mixed failure modes.

Pearson correlation tests initially revealed a poor correlation (-0.122) when all groups were included in the analysis. However, upon closer examination of the data, it became evident that the HFs groups exhibited different behaviors compared to the SP30 and P30 surface treatments. A subsequent Pearson correlation test excluding the HFs groups showed a moderate negative correlation (-0.418) between shear bond strength and contact angle. This suggests a tendency for increased shear bond strength as the contact angle decreases.

## Discussion

The failure rate of resin composite restorations ranges between 1-4% annually and can account for up to 60% of the demand for procedures in a general practice, primarily due to issues with marginal integrity and secondary decay. Repairing such restorations usually consists in a more conservative and cost/time effective solution, which contributes to prevent deterioration of the tooth and the progressive compromise of remaining tooth structure [4-14].

There is a lack of consensus regarding repair protocols, particularly considering the variations in resin composite formulations. However, it is evident that successful repair depends on the chemical and/or mechanical interaction between fractured and repairing resins [3-6,8,11,13-15]. Therefore, the resin composite surface should be treated using a protocol capable of removing degraded superficial resin and increasing surface roughness. This can be achieved through surface roughening techniques such as sandblasting, diamond burs, or abrasive discs, as well as chemical treatments involving acids, silane, and adhesive systems [3-5,7,10,11,13-22].

Based in the present results, both null hypotheses were denied as the surface treatments and time point for repair changed the interface between base and repair resins.

This study observed a tendency of lower shear bond results for immediate samples treated with hydrofluoric acid (group HFs). This effect may be attributed to the acid’s capability of dissolving filler particles [16], exposing a larger area of resin matrix to hydrolytic degradation, especially in a recently polymerized resin composite [23]. Interestingly, for aged samples, treatment with hydrofluoric acid resulted in similar outcomes to other treatments, and this could be explained by a fully polymerized polymer before acid etching, which prevents the acceleration of hydrolytic degradation, and indicating that the hydrofluoric acid was effective in increasing surface roughness, as reported in previous studies [16].

The results for contact angle support this explanation, as hydrofluoric acid tended to result in lower contact angles for immediate samples compared to aged samples. However, it is noteworthy that for ADM, hydrofluoric acid had detrimental effects on both immediate and aged samples, which may be explained by its different organic composition based in an organic modified ceramic (Ormocer).

While strong acids have been recommended and can yield adequate bonding results depending on the material, they also pose risks for patients, such as soft tissue damage [16,19]. Therefore, the authors of this study do not find a compelling reason to adopt such a protocol. Surface roughening using diamond burs or abrasive discs, although requiring additional clinical steps, can increase surface roughness while reducing risks for patients and without requiring additional equipment such as air abrasion [4,5,14,16,19].

In general, the highest shear bond results were observed with treatments involving mechanical surface roughening, either alone or in combination with a silane agent (groups SP30 and SP30s), which is consistent with findings in the literature [5-7,10,14,22]. The range of shear bond strength values are in agreement with recent literature reporting 20-30 MPa in groups repaired with surface roughening and application of Scotchbond Universal [13].

The results of fracture mode analysis indicated that samples treated with mechanical roughening and/or silane agent (P30s, SP30, and SP30s) predominantly exhibited cohesive and/or mixed failure modes. Furthermore, samples from groups SP30 and SP30s generally exhibited a decrease in contact angle for aged samples compared to other surface treatment methods. This could contribute to enhancing the interaction between the base resin and the adhesive system. However, for most aged resin composites, groups SP30 and SP30s did not show significantly higher shear bond strength compared to other groups. This may be explained by the universal bonding agent used in the present study (Scotchbond Universal).

Some universal bonding agents, such as Scotchbond Universal, contain a silane agent capable of increasing surface wettability and binding organic and inorganic content [3,11,14,18,19,22]. Additionally, it contains 10-methacryloxydecyl dihydrogen phosphate (10-MDP), an acidulated phosphate monomer with an amphiphilic structure capable of bonding to tooth structures, resins cross linked network and oxide metals [3,6,9,14,22].

The composition of the adhesive system may explain why different surface treatments yielded relatively similar results, as the superiority of some universal adhesive systems has been reported in the literature [3,22,24]. Furthermore, the lack of a clear effect of the application of a silane agent may be explained by the presence of this agent in the bonding agent itself [4,22].

The results also showed a tendency for higher shear bond strengths for aged resins, which may indicate that “older” resin composites present higher surface roughness and favors penetration of the adhesive system. Alternatively, it could suggest that the chemical interaction between the adhesive system and the resin composite is enhanced in a more mature polymer, especially when a silane agent is used [5,22].

It was also noteworthy to observe a tendency for higher shear bond results in high viscosity resin composites. This trend could be attributed to the higher filler content present in these composites, along with their potential interaction with the silane agent and 10-MDP contained in the adhesive system. Additionally, the higher filler content may lead to a lower stress concentration at the interface between the base and the repairing resin, due to a more similar Young’s modulus with the repairing resin used in the present study.

Thus, the present study demonstrates that repairing properties are influenced by the type of resin composite, its age, and the surface treatments applied. However, it appears safe to assert that the majority of repairing procedures will be performed on ‘older’ resin composites. Within this context, simplified protocols, such as P30, may promote adequate bonding when Scotchbond Universal is used. Considering only one bonding agent was used, it is important to note that the results obtained in this study may not necessarily be replicated with other bonding agents due to widely variable compositions. Therefore, until further literature becomes available, repairing protocols using other adhesive systems should incorporate mechanical surface roughening and the application of silane + adhesive, or an adhesive system containing silane in its composition [4-6,22].

One could question the use of only one repair resin, as composite repairs are usually suggested to be performed with the same base resin. Nevertheless, clinicians often face limitations regarding previous treatments and the variety of different resin composites available in their offices. Moreover, it has been reported that the most important factor in composite repair is the surface treatment protocol, suggesting that any resin composite could be used [5,15]. It is also important to mention that the phosphoric acid in the present study was primarily used to reduce surface contamination and increase surface energy rather than to increase the surface roughness, in line with previous reports [3,6,9,10,16,20]. It is also noteworthy this study represents a first step towards understanding the effects of different surface treatment protocols considering different substrates, and further studies should also consider the effects of artificial aging after the repairing procedures, including evaluation of surfaces under scanning electron microscopy (SEM). Future studies should also focus on testing different bonding agents and repairing resins to further explore their effects and optimize repair protocols.

Based on the limitations of this study, shear bond strength is influenced by the type and age of the base resin composite, as well as the surface treatment applied. Despite a tendency for higher results when mechanical roughening is associated with Scotchbond Universal, there is not a clear difference to justify surface roughening in most of the resin composites. Furthermore, most of the surface treatments performed similarly, regardless of the base resin composite.

## Figures and Tables

**Table 1:** List of materials and their composition* (with filler % in weight).

Resin	Composition*
Admira Fusion Xtra (ADM)	Ormocer resin, 84% filler based on silicon oxide
Filtek Supreme Flowable (FSF)	Bis-GMA, Bis-EMA, TEGDMA, 65% filler (0.004 - 10µm - based on silica and zirconia)
Filtek One (FO)	AUDMA, UDMA and 1, 12-dodecane-DMA, 76.5% filler (0.004 to 0.1µm - based on silica, zirconia and ytterbium trifluoride)
Vitra (VIT)	Modified UDMA, TEGDMA, 74% filler (100 - 200nm - based on zirconia)
Filtek Supreme (FS)	Bis-GMA, Bis-EMA, UDMA, TEGDMA, 82% filler (0.004 to 10µm - based on silica and zirconia)
Filtek Bulk Fill Flowable (FBF)	UDMA, BISGMA, Bis-EMA, Procrylat resin, 64.5% filler (0.01 to 5µm - based on silica, zirconia and ytterbium trifluoride)

* According to the data provided by the manufacturer.

**Table 2:** List of treatment groups.

Group	Protocol
HFs (1)	Hydrofluoric acid 120s + silane
P30 (2)	Phosphoric acid 30s
P30s (3)	Phosphoric acid 30s + silane
SP30 (4)	Sof-lex + phosphoric acid 30s
SP30s (5)	Sof-lex + phosphoric acid 30s + silane

**Table 3:** Table Results (std deviation), in MPa, of Shear Bond strength for new resin composites.

PROTOCOL \RESIN	HFs	P30	P30s	SP30	SP30s
FS	26.38 (3.84) Aa	30.89 (5.2) ABa	36.03 (5.7) Ba	28.21 (4.1) ABab	29.13 (2.97) ABa
ADM	16.83 (2.65) ABb	25.28 (5.18) BCab	15.64 (3.57) Ab	30.89 (4.03) Ca	20.44 (3.6) ABbc
FBF	23.45 (3.76) Aa	21.78 (3.15) ABb	23.69 (3.47) Abc	24.39 (2.98) Aab	14.55 (3.02) Bc
FSF	13.7 (1.94) Ab	24.09 (4.24) Bab	25.5 (3.98) Bc	21.04 (3.67) Bb	23.15 (4.26) Bab
ONE	19 (3.76) Aab	30.19 (3.6) BCab	37.64 (6.07) Ba	23.94 (4.32) ACab	26.58 (5.3) ACab
VT	19.71 (2.77) Aab	24.26 (4) Aab	24.64 (3.33) Ac	25.51 (4.27) Aab	23.68 (3.07) Aab

Different uppercase letters mean difference between treatments for the same resin group (*p* <.05) Different lowercase letters mean difference between resins considering the same treatment (*p* <.05)

**Table 4:** Results (std deviation), in MPa, of Shear Bond strength for aged resin composites.

RESIN	HFs	P30	P30s	SP30	SP30s
FS AGED	27.64 (3.38) Aa	23.48 (4.32) Aa	28.61 (4.68) Aa	37.3 (3.41) Ba	25.84 (4.87) Aa
ADM AGED	12.21 (2.73) Ab	24.84 (4.22) Ba	29.38 (4.26) Ba	25.64 (4.89) Bb	24.38 (2.5) Ba
FBF AGED	26.13 (4.34) Aa	22.51 (4.63) Aa	28.35 (5.62) Aa	26.54 (2.75) Ab	27.95 (5.12) Aa
FSF AGED	25.88 (4.51) Aa	22.55 (4.28) Aa	28.04 (3.18) Aa	26.88 (4.39) Ab	23.44 (3.96) Aa
ONE AGED	31.78 (4.55) Aa	28.35 (4.26) Aa	29.91 (5.03) Aa	28.36 (5.33) Ab	29.09 (3.43) Aa
VT AGED	25.36 (4.64) Aa	22.54 (4.08) Aa	30.85 (5.68) Aa	29.19 (5.68) Aab	25.86 (4.47) Aa

Different uppercase letters mean difference between treatments for the same resin group (*p* <.05) Different lowercase letters mean difference between resins considering the same treatment (*p* <.05)

## Data Availability

The datasets used and/or analyzed during the current study are available from the corresponding author.
